# Application of valencene and prospects for its production in engineered microorganisms

**DOI:** 10.3389/fmicb.2024.1444099

**Published:** 2024-08-07

**Authors:** Yafeng Song, Huizhong Liu, Wim J. Quax, Zhiqing Zhang, Yiwen Chen, Ping Yang, Yinhua Cui, Qingshan Shi, Xiaobao Xie

**Affiliations:** ^1^Guangdong Provincial Key Laboratory of Microbial Culture Collection and Application, State Key Laboratory of Applied Microbiology Southern China, Guangdong Detection Center of Microbiology, Institute of Microbiology, Guangdong Academy of Sciences, Guangzhou, China; ^2^Department of Chemical and Pharmaceutical Biology, Groningen Research Institute of Pharmacy, University of Groningen, Groningen, Netherlands

**Keywords:** valencene, valencene synthase, metabolic engineering, terpenoids, synthetic pathway

## Abstract

Valencene, a sesquiterpene with the odor of sweet and fresh citrus, is widely used in the food, beverage, flavor and fragrance industry. Valencene is traditionally obtained from citrus fruits, which possess low concentrations of this compound. In the past decades, the great market demand for valencene has attracted considerable attention from researchers to develop novel microbial cell factories for more efficient and sustainable production modes. This review initially discusses the biosynthesis of valencene in plants, and summarizes the current knowledge of the key enzyme valencene synthase in detail. In particular, we highlight the heterologous production of valencene in different hosts including bacteria, fungi, microalgae and plants, and focus on describing the engineering strategies used to improve valencene production. Finally, we propose potential engineering directions aiming to further increase the production of valencene in microorganisms.

## 1 Introduction

**Figure 1:**
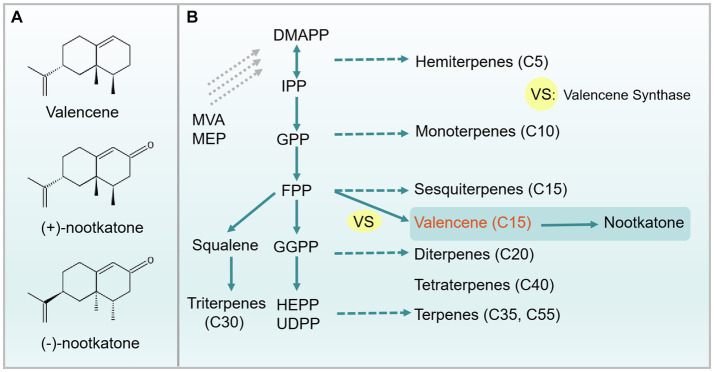
Chemical structure of valencene and nootkatone, and the biosynthesis pathway. **(A)** Chemical structure of valencene, (+)-nootkatone, (−)-nootkatone. **(B)** The biosynthesis pathway of valencene, nootkatone and different types of terpenoids. Metabolite abbreviations: IPP, Isopentenyl pyrophosphate; DMAPP, Dimethylallyl pyrophosphate; GPP, Geranyl pyrophosphate; FPP, Farnesyl pyrophosphate; GGPP, Geranylgeranyl pyrophosphate; HEPP, Heptaprenyl pyrophosphate; UDPP, Undecaprenyl pyrophosphate. Solid lines represent reactions being catalyzed by specific enzymes in one-step, and dashed lines represent reactions being catalyzed by different enzymes or in multiple steps.

Valencene (C_15_H_24_), a carbobicyclic sesquiterpene (Figure 1A), is one of the flavor compounds that possess the odor of sweet, fresh citrus, herb, woody and orange notes (Frohwitter et al., 2014). The molecule gets the name of valencene from the place where it can be most commonly found: Valencia. In fact, valencene is naturally produced by numerous citrus-scented strains and fruits such as grapefruit, tangerine, orange, nectarines, and mangoes (Umano et al., 2002; Hognadottir and Rouseff, 2003; Smit et al., 2019). Moreover, valencene comprises the essential oil components of many medicinal plants, for instance, *Myrica rubra* (Ambroz et al., 2017), *Cyperus rotundus* (Tsoyi et al., 2011), *Alpinia oxyphylla Miq*. (Deng et al., 2024) and so on. Valencene has an immense variety of applications in daily life ranging from food, personal care and home care products (Schempp et al., 2018; Chen et al., 2019). In the beverage industry and in fragrances, natural valencene is extensively used (Schempp et al., 2018). The market value of valencene is estimated to grow to 9.2 million dollars by 2033.^1^

Traditionally, valencene is extracted from oranges, which faces the challenge of low amounts (with only 0.2–0.6% by weight in citrus fruits) and unstable quality due to unpredictable harvest conditions and weather (Beekwilder et al., 2014). Unhealthy growth conditions of citrus trees, including infection of devastating citrus disease, led to the dropped production of oranges as well as the decrease in biosynthesis of valencene and other terpenoids in the fruits (Yao et al., 2019). Recently, producing valencene by fermentation using renewable resources has attracted increasing attention. Isobionics (acquired by BASF in 2019) focuses on developing, producing and selling natural products serving as flavor and fragrance. It was the first company that brought the natural ingredient (Valencene Pure^™^) to the market in 2010,^2^ which was approved as food additive in Japan in September 2020.^3^ Likewise, the USA biotechnology company Evolva can also provide consistent quality valencene^4^ with up to 94% purity (meets the EU Flavor regulation) via sustainable fermentation, which was historically unavailable because of technical restrictions.

In this review, the endogenous biosynthesis of valencene in plants is described, followed by the up-to-date study of valencene synthase. Furthermore, we briefly introduce the effects of valencene on insect repellency and its pharmacological activities. Subsequently, we emphasize the heterologous production of valencene by different hosts and the corresponding engineering strategies, including their possible expansion to other terpenoids. At the end, we propose the possible future engineering directions to further enhance the production of valencene in microbes.

## 2 Biosynthesis of valencene in native hosts

Valencene belongs to the family of isoprenoids, also known as terpenoids, representing a large group of natural products composed of a different number of isoprene precursors (Bohlmann and Keeling, 2008). They can be structurally divided into hemiterpenoids, monoterpenoids, sesquiterpenoids, diterpenoids, sesterterpenoids, triterpenoids, tetraterpenoids, and polyterpenoids, according to the number of five-carbon isoprene units (Figure 1B). Functionally, terpenoids possess a diversity of biological functions including flavors, drugs, and nutraceuticals that are widely used in the food, cosmetic, pharmaceutical, and agricultural industries (Bian et al., 2017).

**Figure 2:**
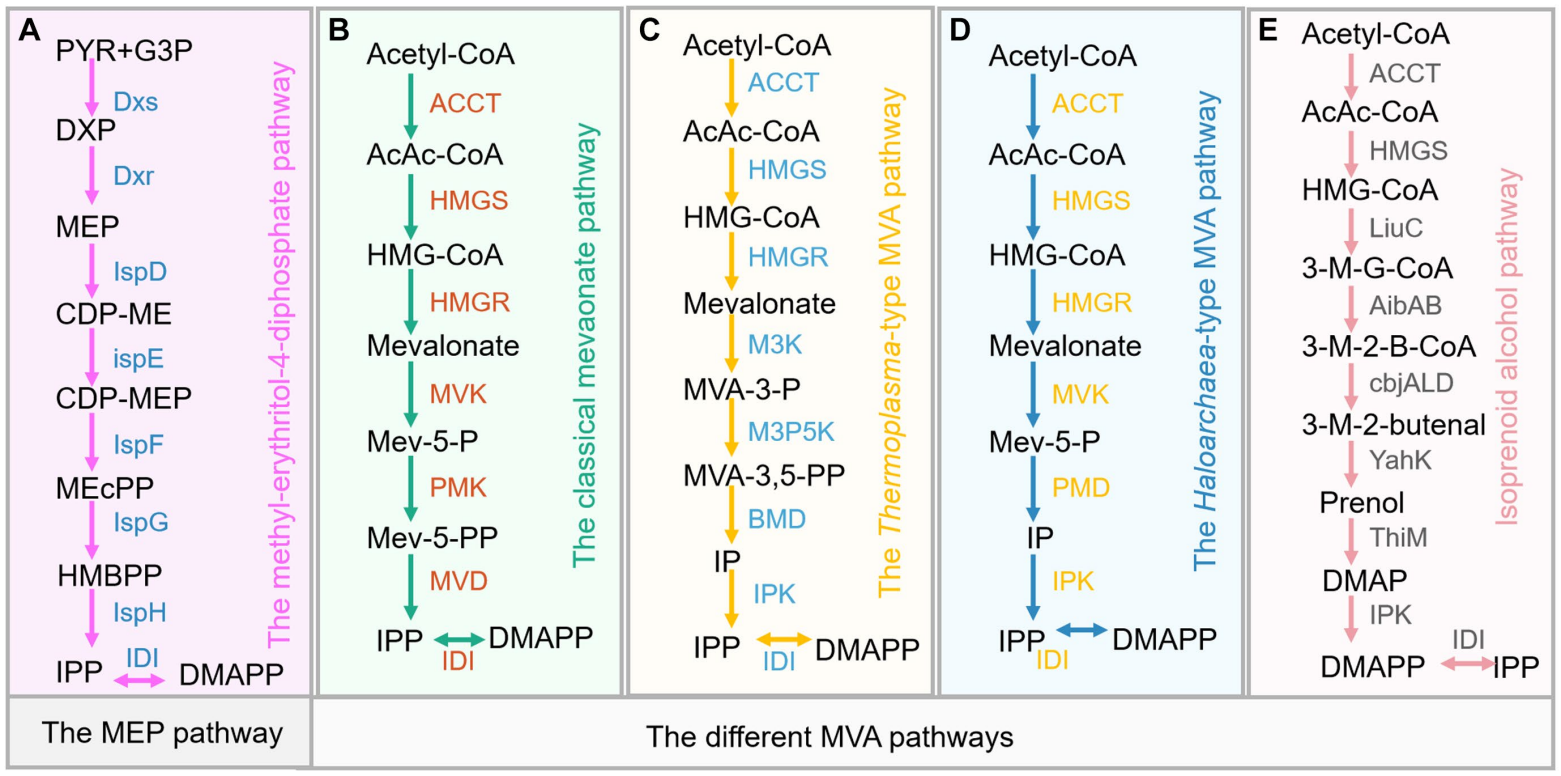
Five types of isoprenoid biosynthetic pathways. **(A)** The 2-C-Methyl-D-erythritol 4-phosphate (MEP) pathway. **(B)** The classical mevalonate (MVA) pathway. **(C)** The *Thermoplasma*-type MVA pathway. **(D)** The *Haloarchaea*-type MVA pathway. **(E)** The isoprenoid alcohol pathway. Dxs, 1-Deoxy-D-xylulose-5-phosphate synthase; IspC, 1-Deoxy-D-xylulose-5-phosphate reductoisomerase; IspD, 4-Pyrophosphocytidyl-2-C-methyl-D-erythritol synthase; IspE, 4-Pyrophosphocytidyl-2-C-methyl-D-erythritol kinase; IspF, 2C-Methyl-D-erythritol 2,4-cyclopyrophosphate synthase; IspG, 1-Hydroxy-2-methyl-2-(E)-butenyl 4-pyrophosphate synthase; IspH, 1-Hydroxy-2-methyl-butenyl 4-pyrophosphate reductase; IDI, Isopentenyl pyrophosphate isomerase; IspA, Farnesyl pyrophosphate synthase; ACCT, acetoacetyl-CoA thiolase; HMGS, 3-hydroxy-3-methyl-glutaryl-CoA synthase; HMGR, 3-hydroxy-3-methyl-glutaryl-CoA reductase; MVK, mevalonate kinase; PMK, phosphomevalonate kinase; MVD, mevalonate-5-diphosphate decarboxylase; IDI, isopentenyl diphosphate isomerase; PMD, phosphomevalonate decarboxylase; M3K, mevalonate 3-kinase; M3P5K, mevalonate 3-phosphate 5-kinase; BMD, bisphosphomevalonate decarboxylase; LiuC, enoyl-CoA hydratase; AibAB, glutaconyl-CoA decarboxylase; cbjALD, acyl-CoA reductase; YahK, alcohol dehydrogenase; ThiM, hydroxyethylthiazole kinase; IPK, phosphomevalonate decarboxylase. Metabolite abbreviations: G3P, Glyceraldehyde-3-phosphate; PYR, pyruvate; DXP, 1-Deoxy-D-xylulose 5-phosphate; MEP, 2-C-Methyl-D-erythritol 4-phosphate; CDP-ME, 4-(Cytidine 5′-pyrophospho)-2-C-methyl-D-erythritol; CDP-MEP, 2-Phospho-4-(cytidine 5′-pyrophospho)-2-C-methyl-D-erythritol; MEcPP, 2-C-Methyl-D-erythritol 2,4-cyclopyrophosphate; HMBPP; 1-Hydroxy-2-methyl-2-butenyl 4-pyrophosphate; AcAc-CoA, acetoacetyl-CoA; Mev-5-P, mevalonate-5-phosphate; Mev-5-PP, mevalonate-5-pyrophosphate; MVA-3-P, mevalonate-3-phosphate; MVA-3,5-PP, mevalonate-3,5-biphosphate; IP, isopentenyl phosphate; 3-M-G-CoA, 3-methylglutacoyl-CoA; 3-M-2-B-CoA, 3-methyl-2-butenoyl-CoA; 3-M-2-butenal, 3-methyl-2-butenal; DMAP, dimethylallyl phosphate. IPP, Isopentenyl pyrophosphate; DMAPP, Dimethylallyl pyrophosphate; GPP, Geranyl pyrophosphate; FPP, Farnesyl pyrophosphate; GGPP, Geranylgeranyl pyrophosphate; HEPP, Heptaprenyl pyrophosphate; UDPP, Undecaprenyl pyrophosphate.

In nature, the biosynthesis of sesquiterpene is realized either via the cytosolic mevalonate (MVA) pathway in eukaryotes or 2-C-methyl-D-erythritol 4-phosphate (MEP) pathway in some bacteria and plastids of plants, as well as alternative MVA pathways in some archaea (Jain et al., 2014; Tholl, 2015). In plants, the MVA pathway starts from acetyl-CoA, which is being converted to isopentenyl pyrophosphate (IPP, C5) through a series of six enzymatic steps (Figure 2). Acetoacetyl-CoA thiolase (ACCT) catalyzes the conversion of acetyl-CoA to acetoacetyl-CoA (AcAc-CoA), and followed by the conversion of AcAc-CoA to MVA by 3-hydroxy-3-methyl-glutaryl-CoA synthase (HMGS) and 3-hydroxy-3-methyl-glutaryl-CoA reductase (HMGR). Next, MVA is sequentially phosphorylated to form mevalonate-5-phosphate (Mev-5-P) and mevalonate-5-pyrophosphate (Mev-5-PP) and then being decarboxylated to generate IPP by the enzymes mevalonate kinase (MVK), phosphomevalonate kinase (PMK), and mevalonate-5-diphosphate decarboxylase (MVD). Subsequently, the isomerase (Idi) catalyzes IPP to form dimethylallyl pyrophosphate (DMAPP, C5). The plastidial MEP pathway involves seven enzymatic steps to form the central isoprene precursor IPP and DMAPP, which starts from the conjugation of glyceraldehyde 3-phosphate (G3P) and pyruvate (PYR) (Figure 2A). Subsequently, farnesyl pyrophosphate synthase condenses DMAPP and IPP into the common precursor of sesquiterpene, farnesyl pyrophosphate (FPP, C15), followed by valencene synthase catalyzing the FPP to valencene (Figure 1B). In addition, valencene can be oxidized to form nootkatone (Figure 1B), which also possesses pleasant odor and interesting therapeutic potentials. Numerous studies investigate catalyzing valencene to form nootkatone by chemical conversion, biotransformation and P450-mediated oxidation in microbial hosts (Kolwek et al., 2018; Li X. et al., 2021; Ye et al., 2022; Zhang et al., 2023). The production, function and heterologous synthesis of nootkatone and its applications have been comprehensively discussed and reviewed by multiple publications (Fraatz et al., 2009; Li X. et al., 2021; Zhang et al., 2023). The valencene productions and related engineering approaches in different hosts are only briefly mentioned. Here, we mainly highlight the recent advance of valencene from a variety of perspectives, hoping to give clear and overall insights into valencene application prospects and engineering strategies.

Valencene can be found in the leaves, peel and oil glands of citrus. However, the level of valencene extracted from peel (12.84 mg/kg) is much higher than from flesh (0.08 mg/kg), as well as other sesquiterpenes in citrus [4]. It is also known that the volatile compound profiles of citrus fruits are different and complex. For instance, the content of valencene in sweet orange was much more abundant than in other citrus species. In the well-known Chinese medicinal plant *Alpinia oxyphylla* Miq, (+)-valencene can be detected in different developmental stages of seed and fruit, but it is predominantly distributed in immature and mature seeds (Deng et al., 2024).

Regarding the production-development stage relationship, valencene is the main sesquiterpene that highly accumulates during fruit development period, with a 9-fold and 93-fold increase observed at 180 days after full bloom (DAFB) and 210 DAFB, respectively (Shen et al., 2016). The expression of the valencene synthase, which is regulated by the citrus transcription factors CitAP2.10, consistently increases. Valencene synthesis is not only affected by the development stages of plants, but also by plant hormone stimuli such as the activation of the ethylene signaling pathway. Valencia orange fruit accumulates over 20% more valencene when treated with ethylene compared to the level of the control group that treated with air (Sharon-Asa et al., 2003). Meanwhile, the transcription level of valencene synthase shows an eight-fold increase compared to the counterpart group. Other conditions such as irrigation are also relevant to the presence of valencene in canopies of *Olea europaea L.* trees (Benelli et al., 2015).

As one of the major terpenes in orange oil, valencene has traditionally been used as an indicator of good quality of orange oil (Elston et al., 2005). When the citrus trees developed Huanglongbing (also known as citrus greening), one of the most severe and devastating citrus diseases, the symptomatic fruits often appeared immature and the had off-flavor characterized as bitter, harsh, sour or metallic. Further investigation discovered that this was associated with a decreased level of valencene and other volatile terpenoid products as well as the reduced expression levels of enzymes in glycolysis, tricarboxylic acid cycle, amino acid biosynthesis and terpenoid biosynthesis pathways (Dala-Paula et al., 2018; Yao et al., 2019). However, an opposite opinion proposed that valencene produced no aroma activity. The role that valencene plays may simply be as a marker for increased fruit maturity, since valencene levels are highly correlated with increased orange flavor quality (Elston et al., 2005). Nevertheless, providing the characteristic aroma of sweet orange flavor, valencene has been extensively used in many fields ranging from food, beverage and cosmetic industries, with a continuously increasing demand.

## 3 A critical enzyme in the valencene biosynthesis pathway: valencene synthase

Valencene synthase and its variants are the key enzymes in synthesizing valencene molecules. Until now, multiple valencene synthases have been identified and validated to be functional enzymes either *in vivo* or *in vitro.* They originated from *Vitis vinifera L.* (VvVal), Citrus paradise (GFTpsE / CitrusVS), *Citrus sinensis* cv. (Cstps1) and *Callitropsis nootkatensis* (CnVS), *Eryngium glaciale* (EgVS), and *Alpinia oxyphylla* (AoVS) (Lucker et al., 2004; Elston et al., 2005; Schalk and Clark, 2008; Beekwilder et al., 2014; Ye et al., 2022; Deng et al., 2024). Valencene synthase belongs to the family of sesquiterpene synthases, which are responsible for converting substrate FPP to different cyclic sesquiterpenoids through complex catalytic mechanisms (Degenhardt et al., 2009). Despite the significant difference in product profile and amino acid sequence among various sesquiterpene synthases, they all share the class I terpene synthase α-helical fold, with two most conserved motifs (“DDXXD/E”-motif, and “DTE/NSE”) (Miller and Allemann, 2012).

But information regarding catalytic parameters of valencene synthases remains limited, except CnVS. Similar to most sesquiterpene synthases, no predicted N-terminal targeting sequences exist in CnVS, implying its cytosolic localization. Additionally, low protein-sequence similarities were observed between CnVS and other reported valencene synthases, for example, 32% sequence identity with CitrusVS (Beekwilder et al., 2014). The robust enzyme CnVS, showed high activity under different pH and temperature regimes, which could potentially broaden the range of applicable hosts and physiological conditions. However, valencene synthases exhibited relatively lower catalytic efficiencies compared to other sesquiterpene synthases, such as *Artemisia annua* amorphadiene synthase, tobacco epi-aristolochene synthase and *Hyoscyamus muticus* premnaspirodiene synthase (Takahashi et al., 2007; Beekwilder et al., 2014; Abdallah et al., 2018).

Sesquiterpene synthases also exhibit catalytic product promiscuity, i.e., most sesquiterpene synthases generally produce multiple products but one of them being the major product. For example, aristolochene synthase from *Penicillium roqueforti* transformed FPP to aristolochene, germacrene and valencene in a 94:4:2 ratio (Felicetti and Cane, 2004). Regarding valencene synthases, products formed by VvVal include valencene and 7-epi-α-selilnene as well as five other minor side products (Lucker et al., 2004; Beekwilder et al., 2014). CnVS can convert FPP to both valencene and germacrene A as dominant products (Chappell, 2004; Beekwilder et al., 2014). The product promiscuity is considered to be the “darker” side of enzyme cross-reactivity/specificity, which is not desired in catalytic processes in industries. Enzyme engineering provides efficient approaches to improving product specificity, thermostability and catalytic efficiency by directed evolution or site-directed mutagenesis (Yoshikuni et al., 2006; Diaz et al., 2011; Zhou et al., 2021). For instance, the EgVS mutant (I533V, R336K, H196R, D176E, R306K, K325E) produced a 3.15 times higher titer of valencene than the wild type EgVS, and the valencene: aristolochene ratio increased from 2.97: 1 to 4.18: 1 (Ye et al., 2022).

## 4 Biological activities

Many terpenoids display various biological activities and have been used for the treatment of human diseases. In recent decades, valencene has attracted increasing scientific interest to reveal its bioactivities and functions as a pharmaceutical entity candidate. Like the majority of terpenes, valencene could keep insects away. Emerging evidence suggests that valencene could be an alternative means of mosquito control due to its insect repellent effects (Tisgratog et al., 2018). Also, valencene has shown a strong repellent effect and obvious contact toxicity against *Tribolium castaneum* adults (red flour beetles), which is comparable to the powerful commercial pesticides DEET (Guo et al., 2019). Moreover, numerous plants have traditionally been used to enhance human health or as therapeutic herbs for treating diseases (Tsoyi et al., 2011; Rahamooz Haghighi et al., 2017; Zhang et al., 2018; Anunciação et al., 2020). Sesquiterpenes comprise the important components of these herbs. Among the natural compounds, valencene has been investigated to display anti-allergic activity (Jin et al., 2011), anti-inflammatory effects (Yang et al., 2016), immunostimulatory effects (Sunitha et al., 2021), and skin protection effects (Choi et al., 2012), indicating its potential application in multiple fields. Further animal studies and preclinical trials are needed to explore and establish the efficacy of valencene for disease prevention and treatment.

## 5 Biosynthesis and engineering of valencene in microbes and plants

Valencene is widely found in natural sources and can be extracted by distillation from plants. However, commercial-scale production of valencene can also be obtained through chemical synthesis, either by catalyzing starting materials, such as epimeric equatorial ester, 4, 4 -ethylenedioxy-2-methylcyclohexanone, or 4β, 4aβ -dimethyl-Δ6, 7-octalin-1-one ethylene acetal, or by reducing (±)-nootkatone into (±)-valencene (Zhang et al., 2023). However, the chemical synthesis of terpenoids faces disadvantages including chiral recognition and determination of enantiomeric composition, which makes production challenging (De Los Santos and Wolf, 2020).

**Figure 3:**
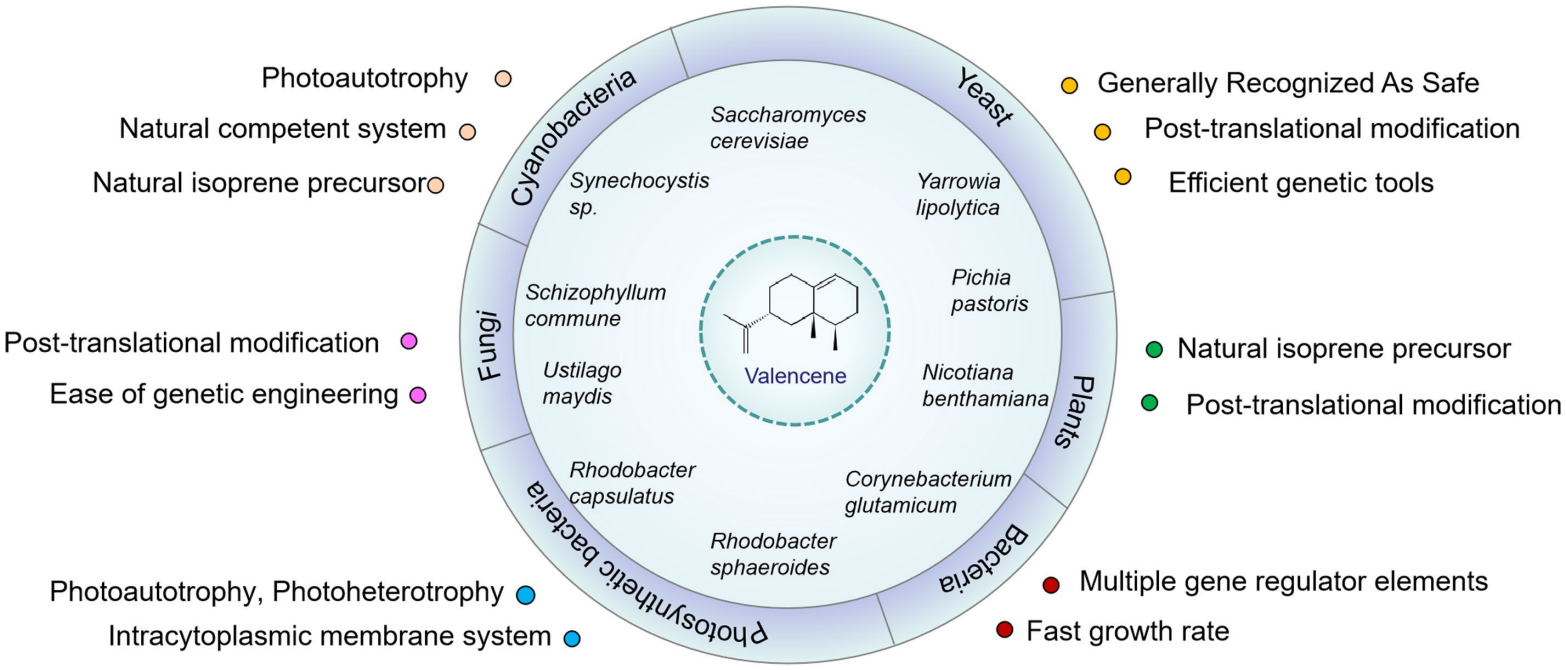
Engineered microorganisms and plants for the production of valencene. Some important advantages of these hosts for the biosynthesis of terpenoids were listed. To highlight the features of photosynthetic bacteria and yeast, they were listed separately with bacteria and fungi, respectively.

An alternative production mode is provided by biotransformation for sustainable production of valencene or nootkatone, as well as by heterologous synthesis in robust and versatile microbial cell factories. In addition, synthetic biology and systematic metabolic engineering can provide the tools and platform for developing and optimizing novel organisms in a more efficient and controllable way (Kavscek et al., 2015). Moreover, harvesting valencene or other interest compounds through fermentation possesses the advantages of scalability, price, and supply flexibility (Lenihan et al., 2008). The currently accessible valencene sources, including such as yeast, fungi, phototrophic bacteria, cyanobacteria and plants (Figure 3), are shown in Table 1. Research has been carried out in various hosts, resulting in produced valencene levels ranging from 1.5 mg/L to 16.6 g/L (Table 1). In the following section, we will analyze the minute aspects of carbon flux in metabolic pathways including the achievements and strategies employed to enhance valencene production.

**Table 1:** Main engineering strategies for improved production of valencene in microorganisms and plants.

Strains	Valencene synthase origin	Engineering strategy	Valencene production	References
*Saccharomyces cerevisiae*	*Eryngium glaciale*	Enhanced MVA pathway, selection of valencene synthase (EgVS), expression of EgVS under the control of GAL promoter, mutation of EgVS (I533V, R336K, H196R, D176E, R306K, K325E), knockout of GAL80, coupling cell growth and biochemical pathway induction Mutation of EgVS increased valencene production from 78.9 ± 11.4 mg/L to 248 ± 4.1 mg/L; Coupling valencene production and cell growth increased its production from 12.4 g/L to 16.6 g/L	16.6 g/L, 120 h, 15 L bioreactor	Ye et al. (2022)
*Saccharomyces cerevisiae*	*Callitropsis nootkatensis*	A 549-fold increase of valencene production was achieved by combining the following strategies: codon optimized valencene synthase CnVS, enhanced MVA pathway, enhanced supply of acetyl-CoA and NADPH, genome integration of three copies of fusion protein ERG20-CnVS (3.2-fold increase). Fed-batch fermentation increased valencene production from 336.4 mg/L to 1.2 g/L	1.2 g/L, 144 h, shake flask	Cao et al. (2022)
*Saccharomyces cerevisiae*	*Callitropsis nootkatensis*	Optimized central metabolism of chassis cell with enhanced supply of acetyl-CoA and NADPH, introduction and optimization of the *Haloarchaea-*Type MVA pathway in yeast peroxisome, genome integration of three copies of valencene synthase CnVS (increased valencene titer from 35.4 mg/L to 73.2 mg/L), overexpressing *PEX28* to increase the number of peroxisomes (increased valencene titer by 50% to 110.6 mg/L), *in situ* integration of auxotrophic markers (*HIS3, URA3*)	869 mg/L, 150 h, shake flask	Cao et al. (2023)
*Saccharomyces cerevisiae*	*Callitropsis nootkatensis*	Evaluation of valencene yields from glucose and mannitol by elementary mode analysis, obtaining strains with mannitol-assimilating ability through adaptive laboratory evolution, enhancing mannitol assimilation by overexpressing the mannitol dehydrogenase and mannitol transporter, enhancing the MVA pathway, decreasing the branch pathways, improving the generation of NADPH, restoring the auxotrophic markers (*TRP1, URA3*), and decreasing fermentation temperature Valencene titer increased by 3-fold when using mannitol instead of glucose as the substrate; High cell-density fermentation of strain with complementing the auxotrophic markers and mannitol feeding increased valencene production from 161.1 mg/L to 5.6 g/L	5.6 g/L, 200 h, 3 L bioreactor	Zhu et al. (2022)
*Saccharomyces cerevisiae*	*Callitropsis nootkatensis*	Overexpression of valencene synthase CnVS, optimized MVA pathway, fusion ERG20 and CnVS with different linkers, knockdown squalene synthesis pathway	217.95 mg/L, 90 h, shake flask	Meng et al. (2020)
*Saccharomyces cerevisiae*	*Callitropsis nootkatensis*	Overexpression of valencene synthase CnVS, different genome sites editing, overexpression of all the MVA pathway genes, screening of suitable promoter-terminator pairs for valencene production	89.7 mg/L, 192 h, shake flask; 539.3 mg/L, 136 h, 3 L bioreactor	Chen et al. (2019)
*Saccharomyces cerevisiae*	*Citrus sinensis*	Overexpression of valencene synthase CsTPS1, N-terminal-truncated tHMG and farnesyl diphosphate synthase (FDPS), targeting CsTPS1 and FDPS to mitochondria by using mitochondrial targeting signal peptides	1.5 mg/L, 6 days, shake flask	Farhi et al. (2011)
*Saccharomyces cerevisiae*	Citrus	Overexpression of valencene synthase CVS by a modified pESC vector, restoration of leucine	20 mg/L, 216 h (9 days), shake flask	Takahashi et al. (2007)
Yarrowia lipolytica (MatA)	*Callitropsis nootkatensis*	Overexpression of valencene synthase CnVS, HMG, ERG12, RG20, and IDI	113.9 mg/L, 72 h, glass tubes	Arnesen et al. (2020)
Pichia pastoris	*Callitropsis nootkatensis*	Overexpression of valencene synthase, premnaspirodiene oxygenase and cytochrome P450 reductase, alcohol dehydrogenase and truncated hydroxy-methylglutaryl-CoA reductase	166 mg/L, 60 h, shake flask	Wriessnegger et al. (2014)
Schizophyllum commune	*Callitropsis nootkatensis*	Overexpression of valencene synthase CnVS, knockout *thn* gene to avoid the production of schizophyllan	16.6 mg/L, 6 days, Erlenmeyer flasks	Scholtmeijer et al. (2014)
*Ustilago maydis*	*Callitropsis nootkatensis*	Fusion of valencene synthase CnVS with GFP at the N-terminus and GFP, fusion of a nuclear export signal to the N-terminus of GFP, deletion of *car2*	5.5 mg/L, 48 h, baffled flasks	Lee et al. (2020)
Synechocystis sp. PCC6803	*Callitropsis nootkatensis*	Overexpression of valencene synthase ValCS and farnesyl diphosphate synthase	9.6 mg/L, 5 days, airtight Hungate tubes	Matsudaira et al. (2020)
Synechocystis sp. PCC6803	*Callitropsis nootkatensis*	Deletions of *shc* and *sqs*, downregulating the expression of *crtE*, overexpression of CnVS-ispA-operon	19 mg/g, 48 h, 6-well plates	Dietsch et al. (2021)
*Rhodobacter sphaeroides*	Nootka cypress (*Callitropsis nootkatensis*)	Overexpression of valencene synthase CnVS with an N-terminal MBP coding sequence, and the mevalonate operon from *Paracoccus zeaxanthinifaciens*	352 mg/L, 3 days, shake flask	Beekwilder et al. (2014)
*Rhodobacter capsulatus*	*Citrus sinensis* *Callitropsis nootkatensis*	Overexpression of valencene synthase CnVS and farnesyl diphosphate synthase	18 mg/L, 24 h, Hungate tubes	Troost et al. (2019)
*Corynebacterium glutamicum*	*Citrus sinensis* *Callitropsis nootkatensis*	Overexpression of valencene synthase CnVS and farnesyl diphosphate synthase	2.41 mg/L, 48 h, shake flasks	Frohwitter et al. (2014)
*Corynebacterium glutamicum*	*Callitropsis nootkatensis*	Overexpression of codon-optimized valencene synthase oCnVS under the control of the light-mediated optimized lac promoter, overexpression of farnesyl diphosphate synthase and isopentenyl pyrophosphate isomerase	41 mg/L, 48 h, oxygen-unlimited FlowerPlate cultivations	Binder et al. (2016)

### 5.1 Engineering of yeast for valencene production

**Figure 4:**
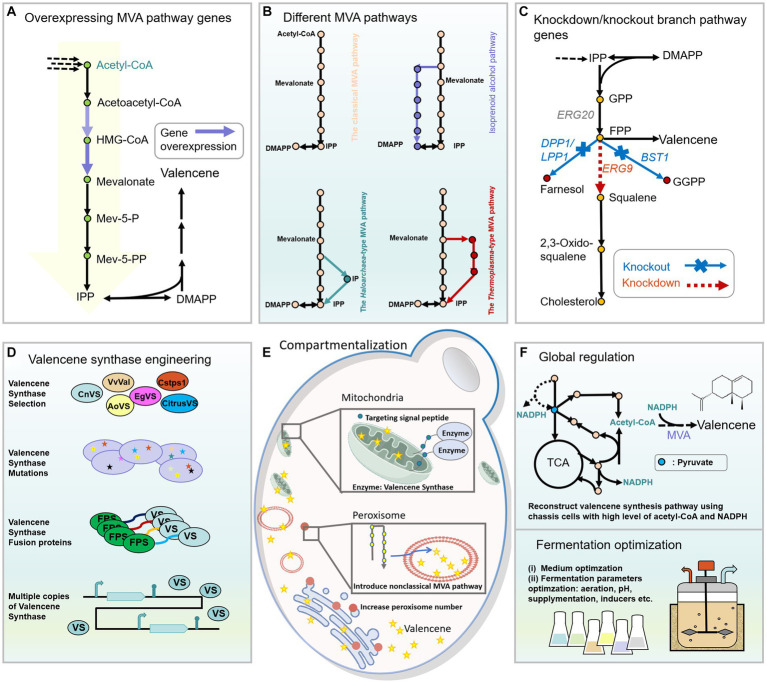
Engineering strategies used to improve the production of valencene in yeast. **(A)** Overexpressing rate-limiting enzymes of the MVA pathway. **(B)** Employing different MVA pathways to reconstruct the biosynthetic pathway of valencene in peroxisomes of yeast. The detailed information of different MVA can be obtained from Figure 2. **(C)** Decreasing the competing pathways of valencene by knockout nonessential genes or knockdown essential genes in branch pathways. **(D)** Engineering of valencene synthase to improve valencene production by selecting suitable valencene synthases from different origins, creating mutants with high catalytic efficiency or increased product specificity, constructing fusion proteins with various linkers, and fine-tuning the expression levels of valencene synthases. **(E)** Compartmentalization strategies for improving synthesis of valencene: expressing valencene synthases in mitochondria by fusing the mitochondrial targeting signal peptide and reconstructing the valencene synthesis pathway in the peroxisome of yeast. **(F)** Improving valencene production by engineering the global regulation of yeast and optimizing the fermentation process.

Yeast has been considered one of the most predominant hosts for terpenoid production given its generally recognized as safe (GRAS) status, powerful MVA pathway for isoprene precursor synthesis, suitable eukaryotic protein expression system, post-translational modifications, and favorable physiological properties for fermentation (Bureau et al., 2023). The unicellular yeast *Saccharomyces cerevisiae* is one of the frequently used microorganisms in biotechnology with highly appreciated applications in producing both bulk and fine chemicals (Paddon and Keasling, 2014; Baptista et al., 2021). The methylotrophic yeast *Pichia pastoris* can assimilate methanol as the sole carbon source for growth, with an outstanding capacity for producing recombinant proteins and synthesizing numerous compounds ranging from terpenoids to polyketides (Schwarzhans et al., 2017; Pena et al., 2018). The advantage of the oleaginous yeast *Yarrowia lipolytica* as a promising chassis cell for terpenoids production is the high level of lipid and rich intracellular supply of acetyl-CoA (Ma et al., 2019; Li Z. J. et al., 2021). The above-mentioned yeast strains have all been investigated to produce valencene (Table 1). Here, we comprehensively discuss the genetic modifications being modulated to improve the synthesis of valencene (Figure 4).

#### 5.1.1 MVA pathway optimization

Multiple studies have shown the effectiveness of enhancing terpene biosynthesis by overexpressing key MVA pathway enzymes. (i) HMG-CoA reductase (HMGR), catalyzes HMG-CoA to form mevalonate. Overexpression of the soluble N-terminal truncated HMGR (tHMGR) helps release the feedback inhibition of mevalonate on tHMGR, and improves the valencene production to different extents (Farhi et al., 2011; Cao et al., 2022; Ye et al., 2022) (Figure 4A). The feedback inhibition can also be avoided by introducing heterologous and efficient acetoacetyl-CoA thiolase/HMG-CoA reductase gene (EfmvaE), together with HMG-CoA synthase gene (EfmvaS) that originated from *Enterococcus faecalis* (Cao et al., 2023). However, sometimes merely overexpressing tHMGR might severely inhibit the growth of the host cells resulting in very low level of valencene due to IPP and DMAPP toxicity (Meng et al., 2020). But this decreased titer could be recovered by simultaneously overexpressing Erg20 (Meng et al., 2020), heterologous FDPS (*A. thaliana* or human-derived genes, AtFDPS, HsFDPS) (Farhi et al., 2011), as well as Idi (encodes isomerase that catalyzes IPP to form DMAPP) (Cao et al., 2022), transforming DMAPP and IPP into nontoxic products. Alternatively, valencene synthase EgVS and its mutants with high catalytic efficiencies, could avoid the accumulation of undesired intermediates by immediately transforming them into final products when tHMGR was overexpressed (Ye et al., 2022). (ii) ERG20 is responsible for combining IPP and DMAPP to form GPP and FPP. Overexpressing ERG20 or the fusion protein of ERG20 with valencene synthase benefits valencene production and has been well investigated (Chen et al., 2019; Meng et al., 2020; Cao et al., 2022, 2023; Ye et al., 2022).

The MVA pathway also supplies the necessary building blocks for the synthesis of essential membrane components and important cell metabolites (Figure 4C), including ergosterol, farnesol and ubiquinone, etc. (Jiang et al., 1995; Kuranda et al., 2010). Attenuating or blocking the carbon flux to those branch pathways has been tested to improve FPP availability for more terpenoid biosynthesis (Asadollahi et al., 2008). Squalene synthase (ERG9) condenses two molecules of FPP to form squalene for ergosterol synthesis. A downregulated promoter P_HXT1_ was employed to weaken the ERG9 expression, which sharply increased (+)-valencene titer by 2.3-fold compared to the starting strain (Cao et al., 2022). DPP1 encodes diacylglycerol pyrophosphate phosphatase, which transforms FPP into Farnesol (Farhi et al., 2011). BTS1, which encodes geranylgeranyl diphosphate synthase in *S. cerevisiae*, utilizes FPP as substrate for ubiquinone biosynthesis (Jiang et al., 1995). Indirectly, multiple rate-limiting enzymes of the MVA pathway were integrated into the genome at the locus of the unessential genes (DPP1, BST1) of the branch pathways, and increased the production of valencene by up to 2.9-fold at most (Chen et al., 2019).

Besides the classical MVA pathway, several nonclassical isoprenoid biosynthetic pathways have also been established and engineered to improve the production of valencene in yeast, developing a novel platform for a repertoire of terpenoid synthesis (Figure 4B). Two alternative MVA pathways that were discovered in archaea are used to provide precursors of functional branched-chain lipids, including the Haloarchaea-type MVA pathway and the Thermoplasma-type MVA pathway (Dellas et al., 2013; Azami et al., 2014) (Figure 4D). Another isoprenoid alcohol pathway is an artificially designed non-natural isoprenoid biosynthesis pathway (Clomburg et al., 2019). In this perspective, Cao et al. heterologously expressed the three noncanonical pathways to produce valencene in *S. cerevisiae* peroxisomes compared with the classical MVA pathway. The result indicated the Haloarchaea-type MVA pathway to be the most efficient pathway for (+)-valencene synthesis. Further optimization of the Haloarchaea-type MVA pathway and downstream valencene synthase expression increased the production of valencene to 869 mg/L under fed-batch fermentation (Cao et al., 2023).

#### 5.1.2 Compartmentalization engineering

It is well known that organelles and membrane structures play various important roles in terpenoid biosynthesis and storage in eukaryotes (Chen et al., 2023). Recent studies have demonstrated compartmentalization to be an effective strategy for regulating terpenoids production. This is attributed to the suitable physicochemical environments, facilitating the catalytic reaction of enzymes residing in specific subcellular compartments, while also reducing the harmful effects caused by the accumulation of toxic substances (Jin et al., 2022).

The semi-autonomous organelle mitochondria was selected as a feasible space for valencene biosynthesis considering its higher acetyl-CoA (crucial MVA pathway precursor) content than the cytoplasm (Figure 4E). The expression of valencene synthase fused with the mitochondrial targeting signal peptide (mtCsTPS1) resulted in a three-fold increase of valencene titer compared to strains that expressed cytosolic CsTPS1. When both heterologous FPP synthase and valencene synthase were targeted into mitochondria, along with the overexpression of truncated HMGR, an eight-fold increase in valencene titer was achieved in *S. cerevisiae* (Farhi et al., 2011). In another study, Cao et al., demonstrated that peroxisome to be an ideal subcellular organelle to release the potential of the non-classical isoprenoid biosynthetic pathways for valencene synthesis. Further overexpression of Pex28p, which encoded the membrane protein of peroxisomes, expanded the number of peroxisomes and thus led to a 50% increase in (+)-valencene titer compared with the parent strain (Cao et al., 2023). These studies provide insight into harnessing subcellular organelles for terpenoid synthesis. In addition, the endoplasmic reticulum (ER) and lipid droplets (LDs) are speculated to play crucial roles in terpenoids synthesis and storage, respectively (Jin et al., 2022). With increasing knowledge of the mechanisms underlying ER size expansion and LDs merging, mining novel ways to regulate the size of ER and LDs can potentially improve terpenoid yields.

#### 5.1.3 Valencene synthase engineering

Terpene synthases are pivotal enzymes for terpenoid biosynthesis (Whitehead et al., 2023). Fine-tuning the expression cassette of valencene synthase is an efficient approach to increase valencene production by selecting suitable promoters and terminators (Figure 4D), since they both influence the transcription level of the regulated enzyme. Chen and colleagues constructed a promoter-terminator library consisting of 7 combinations for screening, and obtained the best valencene yield using P_HXT7_-*VS*-T_TPI1_ pair in *S. cerevisiae*. When the optimal P_HXT7_-*VS*-T_TPI1_ expression cassette was further transformed into the FPP accumulated host strain, a 2.7-fold increment of valencene production was observed compared with the control strain, reaching 22.7 mg/g DCW (Chen et al., 2019). Moreover, fusion of valencene synthase with FPP synthase can improve terpenoids production by bringing enzymes close for the efficient cascade of catalysis reactions (Zhou et al., 2021;Cao et al., 2022, 2023) (Figure 4D). Of note, the orders of the two enzymes and sequences of flexible linkers significantly affect the final valencene titers, screening the suitable combination of fusion protein components remains necessary (Meng et al., 2020; Cao et al., 2022). The expression level of terpene synthase might be a bottleneck when a high metabolic flux toward FPP biosynthesis occurs. Therefore, integration of multiple copies of valencene synthase into the genome of yeast assists in releasing the potential of the optimized precursor pathway (Figure 4D). The integration of two and three copies of valencene synthase or ERG-*VS* fusion protein into the genome of *S. cerevisiae* resulted in much higher production of valencene compared with the fourth copy integration (Cao et al., 2022, 2023).

Knowledge-driven protein engineering helps improve enzyme performance in terms of catalytic activity, specificity and evolutionary fitness (Sinha and Shukla, 2019). First, mutant candidates can be predicted using information from protein crystal structures or computer simulations, as well as from the multiple sequence alignment result of the target enzyme and its orthologues. A common approach is to modify the shape of the enzyme reaction cavity through site-directed mutagenesis, allowing it to better accommodate the substrate for binding (Figure 4D). I553V of EgVS has been speculated to increase the volume of the binding cavity based on a simulated three-dimensional model built using the crystal structure of tobacco 5-epi-aristolochene synthase (4RNQ) (Ye et al., 2022). In addition, the distal residues of the enzyme sequence might impact enzyme stability through long-range dynamic conformation changes, leading to more flexible active sites for FPP binding and thus increased activity (Yu and Dalby, 2018). A highlighted example was the EgVS mutant (I533V, R336K, H196R, D176E, R306K, K325E) with mutated residues located either close or far from the binding cavity, resulting in 3.15 times higher level of (+)-valencene than wildtype EgVS (Ye et al., 2022). Meanwhile, the combined favorable mutations of EgVS also increased the ratio of product to by-product (valencene: aristolochene) from 2.97: 1 to 4.18: 1.

#### 5.1.4 Global regulation

To improve terpenoids production, an underexplored area is to rewrite the global cellular metabolism and optimize cell robustness. It has been reported that ROX1 is a transcriptional factor, which can inhibit the expression of hypoxia-induced genes in the MVA pathway and influence ergosterol biosynthesis (Henry et al., 2002). Knockdown of ROX1 improved the yields of valencene more than other single mutants, including shutting down GGPP synthesis and repressing squalene formation. Providing enough acetyl-CoA and NADPH can also improve the (+)-valencene production, as they provide key precursors of the MVA pathway and reducing power, respectively. Cao and colleagues previously constructed a chassis for the overproduction of free fatty acids by comprehensively engineering the central metabolism to ensure an abundant supply of acetyl-CoA and NADPH. To fully utilize this chassis strain with cellular cofactor equilibrium, they reconstructed the valencene biosynthesis pathway, optimized the expression of valencene synthase and achieved a final production of (+)-valencene at 1.2 g/L through fed-batch fermentation in shake flasks (Cao et al., 2022) (Figure 4F). Reprogramming the central metabolism through the introduction of heterologous pathways, fine-tuning the tricarboxylic acid cycle and pentose phosphate pathway, and co-factor engineering are promising directions to further enhance the microbial potential for increased valencene production (Meadows et al., 2016; Wu et al., 2020; Cao et al., 2022).

#### 5.1.5 Fermentation

Metabolic pathway optimization includes both genetic modification and fermentation optimization, as well as their synergistic combination (O’Connor, 2016). Auxotrophic markers play important roles in constructing hyperproducers, but give an undesired phenotype during fermentation (Cao et al., 2022, 2023). The auxotrophic markers (HIS3, URA3) of the prototrophic *S. cerevisiae* strains were restored to simplify the valencene production process by avoiding amino-acid supplementation. Likewise, GAL80 was knocked out to activate the GAL promoter that regulates the expression of valencene synthase in the absence of galactose, which surprisingly increased the titer of (+)-valencene from 393.3 ± 40.6 mg/L to 515 ± 0.36 mg/L (Shi et al., 2019; Ye et al., 2022). Regarding fermentation optimization, multiple proof-of-concepts have attested to the usefulness of improving product yields by using various carbon sources (Singh et al., 2016) (Figure 4F). (+)-valencene production of *S. cerevisiae* CCV22 reached 336.4 mg/L when using YPD medium with rich components, compared to 165.7 mg/L when cultured in minimal media (Cao et al., 2022). By providing rich dissolved oxygen and a higher level of glucose, the production of valencene by *S. cerevisiae* BJM-45 sharply increased from 89.7 mg/L in shake flask to 539.3 mg/L in bioreactor.

Of note, it is necessary to avoid microbe degeneration of the phenotype during long-term industrial fermentation, especially for strains with heavy metabolic burden due to multiple genetic modifications (Wu et al., 2016; Lv et al., 2020). The occurrence of non-productive lycopene strains was speculated to be due to the breakdown of the GAL system, which inhibited the transcription of lycopene biosynthesis pathway genes. Therefore, coupling the production of critical substances for host growth to target compound biosynthesis helps to preserve the normal operation of the GAL system (Liu et al., 2016). The uracil-deficient *S. cerevisiae* strain requires URA3 expression for normal growth (de Souza et al., 2020). When both URA3 expression and valencene synthesis pathway genes were controlled under the GAL promoter (downstream genes are activated in the absence of glucose, and repressed in the presence of glucose), strains with a failed GAL system would not grow regularly in long-term fermentation (Zhao et al., 2018). Meanwhile, the URA3 was also regulated by the glucose-inducible promoter P_HXT1_ for cell growth when glucose exists (Ye et al., 2022). Using this strategy, the resulting strain JGH99 significantly improved (+)-valencene titers to 16.6 g/L at 120 h in 15 L bioreactor with the dry cell weight higher than 100 g/L. In contrast, strains JGH78 without coupling apparatus achieved 12.4 g/L of (+)-valencene at 120 h (Ye et al., 2022). Thus coupling cell growth and biochemical pathway induction was demonstrated to be an effective approach to alleviate the degeneration of strains in long-term fermentation. This type of universally applicable strategy can indirectly couple host growth and target compound production, solving the problem of no available biosensors for specific terpenoids.

Rapid development of molecular tools in yeast provides stepping stones for more comprehensive engineering strategies to mine the treasure of microbial cell factories. To further enhance the production of valencene or other terpenoids for industrial applications, researchers could consider about the following directions: including engineering substrate utilization, optimizing cofactor supply, implementing dynamic metabolic engineering, and modulating hydrophobicity to improve terpenoids accumulation capacity or increase terpenoids secretion.

### 5.2 Engineering of other fungi for valencene production

Despite the limited number of efficient genetic tools in fungi hindering the extensive metabolic rewiring of hosts for natural product biosynthesis, *Schizophyllum commune* and *Ustilago maydis* were explored to produce valencene. *S. commune* possessed the advantages of a relatively short life cycle, growth on defined media and relative ease of genetic modification technology. Initially, the volatile sesquiterpenes produced by *S. commune* (the mushroom-forming fungi, basidiomycete) were demonstrated to be biologically active compounds against wood-decay fungi, indicating the potential of *S. commune* in producing terpenes (Wirth et al., 2021). Further genomic analysis of *S. commune* revealed the functionality of the evolutionarily conserved MVA pathway (Scholtmeijer et al., 2014). When Scholtmeijer et al. introduced CnVS into this fungus, (+)-valencene was detected in both mycelium and fruiting bodies. However, the secretion of high levels of polysaccharide schizophyllan hampered (+)-valencene isolation and purification process (Fowler and Mitton, 2000). Mutation of the *thn* gene significantly decreased schizophyllan secretion, and resulted in a 4-fold increase of (+)-valencene, reaching 16 mg/L (Scholtmeijer et al., 2014) (Table 1).

*U. maydis*, a close relative of higher basidiomycetes, is a promising platform for basic research and production of bioproducts including itaconic acid, biosurfactants and heterologous proteins (Teichmann et al., 2007; Sarkari et al., 2014; Becker et al., 2020). The KEGG pathway database facilitated the discovery of the conserved MVA pathway in *U. maydis* (Lee et al., 2020). The introduction of the fusion protein (the green fluorescent protein at the N-terminus of CnVS) into *U. maydis* and subsequent enhancement of the cytoplasmic localization of the fusion protein, showed a titer of 5.5 mg/L of (+)-valencene in shake flask fermentation (Lee et al., 2020). This initial titer of (+)-valencene in *U. maydis* displayed promising starting point, when compared with the initial titer of 1.3 mg/L in *S. cerevisiae* with CnVS (Cankar et al., 2014). Though higher basidiomycetes are usually difficult to cultivate and require sophisticated genetic tools for gene engineering, *U. maydis* shows a well biomass-degrading ability for alternative carbon sources usage, indicating it worthwhile as next generation bioprocessing platform.

### 5.3 Engineering of phototrophic bacteria for valencene production

The purple non-sulfur photosynthetic bacteria (PNSB) capture light energy and subsequently convert it into chemical energy to support propagation (Zhang et al., 2021). Besides, PNSB can carry out either photoautotrophy, photoheterotrophy or chemolithotrophy, by switching metabolic modes depending on distinct conditions (Basak and Das, 2007). In addition, the abundant intracytoplasmic membrane system of PNSB can serve as storage spaces for accumulated heterologous enzymes and hydrophobic metabolites. Recently, two PNSB, *Rhodobacter capsulatus* and *Rhodobacter sphaeroides*, possessing the native MEP pathway, were investigated to produce heterologous sesqui-, tri- and tetraterpenoids (Khan et al., 2015; Su et al., 2018; Hage-Hulsmann et al., 2021; Wu et al., 2021).

Both *Rhodobacter capsulatus* and *Rhodobacter sphaeroides* can produce valencene by introducing CnVS and further up-regulating precursor supply (Figure 3). Although systematic optimization of the endogenous MEP pathway of these two PNSB has not been well explored, the combination the MEP pathway (the intrinsic MEP pathway or overexpression of the rate-limiting enzymes Dxs and Idi) and the heterologous MVA pathway (overexpression at the genome level) indeed showed a synergic effect on improving valencene production. *Rhodobacter capsulatus*, despite a remarkable 147-fold increase in valencene production observed when overexpressing IspA and the plasmid-containing MVA module, the final titer of valencene only reached 18 mg/L after 5-day cultivation (Troost et al., 2019). However, *Rhodobacter sphaeroides* produced quite an appreciable amount of valencene (354 mg/L) after a 3-day fermentation in a low-cell density culture system at flask-scale. This was achieved by co-overexpressing codon-optimized CnVS with an N-terminus MBP coding sequence and the mevalonate operon from *Paracoccus zeaxanthinifaciens* in plasmids (Beekwilder et al., 2014). It is noteworthy that *R. sphaeroides* has been successfully applied to produce commercial-scale valencene and its oxidative derivative nootkatone marketed by Isobionics (acquired by BASF).^5^ In 2023, the Food Safety Commission of Japan concluded that: “no concern relevant to human health is raised on the food additive “Valencene” produced using *Rhodobacter sphaeroides* 168 strain” (Food Safety Commission of Japan, 2023).

### 5.4 Engineering of *Corynebacterium glutamicum* for valencene production

*C. glutamicum*, a Gram-positive soil bacterium, has been known as a GRAS host for million-ton amino acid production in industry for more than five decades (Tsuge and Matsuzawa, 2021). Moreover, the potential biotechnological applications of *C. glutamicum* have been further expanded by producing diamines, alcohols, and terpenoids (Schneider and Wendisch, 2010; Blombach and Eikmanns, 2011; Heider et al., 2012; Lim et al., 2020). *C. glutamicum* possesses the MEP pathway to naturally produce the long-chain carotenoid C50 decaprenoxanthin (Heider et al., 2012) (Figure 3). Rate-limiting steps of the MEP pathway have been investigated by systematic and combinatorial optimization to improve the precursor pools for terpenoid production (Lee et al., 2020). Nowadays, the terpenoids production profile in *C. glutamicum* has been expanded, including pinene, squalene, astaxanthin, patchoulol, lycopene and so on (Kang et al., 2014; Matano et al., 2014; Henke et al., 2018; Henke and Wendisch, 2019; Park et al., 2019).

However, the inherent MEP pathway did not lead to the formation of valencene with the co-expression of CsTPS1 in *C. glutamicum*, which might be attributed to the lack of FPP catalyzed by endogenous prenyltransferases. Thus, the expression of heterologous FPP synthases was tested (either IspA originated from *E. coli* or ERG20 from *S. cerevisiae*), resulting in the production of valencene. The combination of IspA from *E. coli* and CnVS in *C. glutamicum* produced 2.41 mg/L (+)-valencene after 48 h fermentation (Frohwitter et al., 2014). However, (+)-valencene negatively affected the growth of *C. glutamicum*, as the transcription levels of more than 50 genes altered up to 100-fold when treated with 4 mM (+)-valencene. Fortunately, simultaneous *in situ* extraction of extra (+)-valencene by n-dodecane overlay could significantly reduce the influences on both growth and the global gene-expression profile. To further release the growth-inhibition caused by (+)-valencene, a novel and creative light-controlled expression system was applied to regulate the expression of valencene synthase (Binder et al., 2016). In this system, the 6-Nitropiperonyl-photocaged IPTG-based light induction allowed homogeneous induction of the target gene gradually with an enlarged dynamic control range compared to conventional IPTG induction. In addition, with the codon-optimized CnVS (cCnVS) and overexpression of Dxs (which has been demonstrated to be the first rate-limiting enzyme of the MEP pathway) and Idi (which catalyzes IPP to form DMAPP), as well as the oxygen-unlimited FlowerPlate cultivations, the final (+)-valencene titer in *C. glutamicum* increased approximately 6-fold, reaching 41 mg/L.

### 5.5 Engineering of cyanobacteria for valencene production

Cyanobacteria are solar-powered sustainable cell factories, which can grow photoautotrophically and convert sunlight and CO_2_ into valuable fine chemicals or secondary metabolites (Cheng et al., 2023) (Figure 3). *Synechocystis* sp. PCC 6803 is a natural terpene producer and represents a promising platform for valencene production through cautious carbon flux rewriting due to competing branch pathways (Matsudaira et al., 2020; Dietsch et al., 2021). In this cyanobacterium, two major branch pathways consume FPP. Squalene synthase (*sll0513*, *sqs*) is responsible for the conversion of FPP to squalene, which is further transform into hapanoid by squalene hopane cyclase (*slr2089, shc*) (Englund et al., 2014). The expression of the essential gene *crtE*, catalyzes the consecutive condensation of precursors IPP and DMAPP to GPP, FPP, and finally GGPP for the synthesis of carotenoid (Lin et al., 2017). The double knockout of *sqs* and *shc* led to a metabolic flux shift from FPP toward GGPP and an increased content of inherited carotenoids. Then by overexpressing the CnVS under the rhamnose-inducible promoter, the double-mutant showed a 40% increase in valencene accumulation. Conditionally repressing the crtE and introducing farnesyl pyrophosphate synthase (*ispA*) from *E. coli* pulled FPP from increased carotenoid content to valencene production (Lin et al., 2017). Further construction of IspA and CnVS as an operon accumulated a higher level of valencene (3.5-fold) than only with CnVS or the fusion protein of IspA and CnVS (1.7-fold) (Dietsch et al., 2021). Overall, the multi-component engineering approach highly increased the final production of valencene to 19 mg/g after 48 h cultivation of engineered *Synechocystis* sp. PCC 6803 (Dietsch et al., 2021). These discoveries proved the possibility of redirecting the metabolic flux of terpenoid precursors to produce more desired compounds in cyanobacteria. In addition, the native MEP pathway enzymes of *Synechocystis* sp. PCC 6803 have not been systematically explored or optimized to boost the precursor supply, and this is supposed to be an efficient strategy to further improve the production of valencene or other terpenoids.

### 5.6 Engineering of plants for valencene production

Initiated from the late 1990s, plants have also been deployed to serve as terpenoids production platforms to match the market demands for large amounts of valuable natural products, such as artemisinin and triterpenes (Zhang et al., 2011; Wu et al., 2012). The fast growing tobacco plant *Nicotiana benthamiana* was investigated to produce various terpenoids, including monoterpenoids, sesquiterpenoids, diterpenoids, triterpenoids, and sesterterpenoids etc. (Figure 3). Strategies used to improve the production of target terpenoids mainly involve enhancing the precursor synthesis pathway and producing terpenoids in different subcellular compartments (Reed and Osbourn, 2018). Regarding (+)-valencene, a transient system was set up by introducing the valencene synthase from *Nootka cypress* into the plant host and harvested 0.70 ± 0.13 μg (+)-valencene per gram leaf per 24 h. Decreasing the competing branch pathways by silencing the endogenous squalene synthase and 5-epi-aristolochene synthase increased the production of (+)-valencene by 2.8-fold. However, when two rate-limiting enzymes (the truncated 3-hydroxy-3-methylglutaryl-CoA reductase and farnesyl diphosphate synthase) in the FPP synthesis pathway were overexpressed, further increase of (+)-valencene formation was not observed. This might be caused by the breakdown of FPP leading to the formation of farnesol (Cankar et al., 2015). Though the development of plant production systems remains limited, this investigation indicated the availability of engineering plants to produce valencene. Future directions on developing *Nicotiana benthamiana* as terpenoids host can focus on the large-scale isolation of target compounds and avoiding side-products due to the modification of the target compound by oxidation, glycosylation or dephosphorylation (Reed and Osbourn, 2018).

### 5.7 Future perspectives

Taken together, many achievements have been made to construct valencene-producing microorganisms, but the production is still lower as compared to other sesquiterpenes (Shukal et al., 2019). Therefore, improving valencene production in microbial chassis and expanding its applications remains a promising field that requires further exploration and development. With the rapid advances in cutting-edge techniques, genetic modification of potential strains is more feasible and efficient, thus speeding up the “Design-Build-Test-Learn” cycles. Several suggestions have been proposed to further boost the production of valencene. (i) From the perspective of the complicated metabolic network, the engineering strategies aim at balancing valencene production and cellular growth. This requires the hosts to provide sufficient precursors, eliminate feedback inhibitions, guarantee the availability of cofactors, and efficiently export the products. Since the canonical terpenoid precursors supply often faces the challenge of native competing metabolic networks, the introduction of the isopentenol utilization pathway provides a universal and effective platform for the shortcut synthesis of IPP and DMAPP (Ma et al., 2023). In addition, the underdeveloped subcellular compartmentalization of terpenoid biosynthesis implies promising potential for valencene production due to its unique organelle microenvironments (Yao et al., 2023). Possibly, dual regulation of cytoplasmic and subcellular metabolism is expected to further boost valencene production (Kong et al., 2023). Furthermore, employing exporter-mediated product secretion has been demonstrated to be effective approach to increase terpenoids production through attenuating metabolic burden caused by terpenoids accumulation (Xu et al., 2023). (ii) As the metabolic gatekeeper, valencene synthase determines the flux from precursors to the final products. To take full advantage of this enzyme, it is important to decode its catalytic mechanisms, including deciphering the role of catalytic motifs, active sites, polar residues and metal factors, etc. (Whitehead et al., 2023). Insight into the valencene synthase mechanistic properties helps to accelerate the rational design process, thus improving its catalytic efficiency, as well as understanding and overcoming the complexity and promiscuity of the reactions. (iii) Meanwhile, high-throughput multi-omics technologies provide vast amounts of data and numerous potential genetic targets to improve terpenoids production. Artificial intelligence and machine learning enable the conversion of data into useful knowledge for a better understanding of microbial systems and improving system behaviors (Shi et al., 2022). (iv) For the validation of potential engineering targets, the development of designable and multiplexable genetic tools, the application (DNAda) capable of writing automation instructions for combinatorial DNA assemblies (Nava et al., 2023), and the high throughput automated platform (PlasmidMaker) for plasmid construction (Enghiad et al., 2022) can greatly accelerate the rise of microbe cell factories to produce large-scale valencene.

## 6 Conclusion

Traditionally, valencene is known as a flavor compound and extensively used in the beverage industry and in fragrances. Scientific researches indicate that valencene not only shows biological effects on insects, but also possesses various beneficial pharmacological properties. Due to its biological activities and applications in commercial formulations, valencene shows continuously increasing global market demand. Therefore, it becomes an attractive target for biosynthesis and biotechnological production. This review highlights many achievements that have been made to improve the production of valencene by microorganisms and plants, including yeast, fungi, phototrophic bacteria, cyanobacteria and tobacco. Also, the metabolic engineering strategies to improve valencene production are comprehensively discussed and some future perspectives are proposed, hoping to inspire researchers to further explore and develop microbial cell factories for terpenoids production.

## Author contributions

YS: Conceptualization, Funding acquisition, Writing – original draft, Writing – review & editing. HL: Conceptualization, Writing – review & editing. WQ: Conceptualization, Writing – review & editing. ZZ: Writing – review & editing. YCh: Writing – review & editing. PY: Writing – review & editing. YCu: Writing – review & editing. QS: Supervision, Writing – review & editing. XX: Conceptualization, Funding acquisition, Writing – review & editing.

## Footnotes

^1^ https://www.futuremarketinsights.com/reports/valencene-market

^2^ https://www.prnewswire.com/news-releases/isobionics-introduces-natural-valencene-puretm-95768069.html

^3^ https://www.basf.com/jp/en/media/news-releases/jp/2020/09/2020_0914_nootkatone_japan.html

^4^ https://evolva.com/valencene/

^5^ https://shop.basf.com/isobionics/pages/natural-valencene-80/
